# Monitoring SARS-CoV-2 variant transitions using differences in diagnostic cycle threshold values of target genes

**DOI:** 10.1038/s41598-022-25719-9

**Published:** 2022-12-17

**Authors:** Antoni E. Bordoy, Verónica Saludes, David Panisello Yagüe, Gemma Clarà, Laia Soler, Alexia Paris de León, Cristina Casañ, Ana Blanco-Suárez, Mercedes Guerrero-Murillo, Beatriz Rodríguez-Ponga, Marc Noguera-Julian, Francesc Català-Moll, Irina Pey, Maria Pilar Armengol, Maria Casadellà, Mariona Parera, Raquel Pluvinet, Lauro Sumoy, Bonaventura Clotet, Montserrat Giménez, Elisa Martró, Pere-Joan Cardona, Ignacio Blanco

**Affiliations:** 1 Northern Metropolitan Clinical Laboratory, Microbiology Department, Hospital Universitari Germans Trias i Pujol (HUGTiP), Badalona, Spain; 2 Fundació Institut d’Investigació en Ciències de la Salut Germans Trias i Pujol (IGTP), Badalona, Spain; 3 CIBER in Epidemiology and Public Health (CIBERESP), Madrid, Spain; 4 Clinical Genetics Department, Northern Metropolitan Clinical Laboratory, Hospital Universitari Germans Trias i Pujol (HUGTiP), Badalona, Spain; 5 Institut de Recerca de la SIDA-IrsiCaixa, Hospital Universitari Germans Trias i Pujol (HUGTiP), Badalona, Spain; 6 CIBER in Respiratory Diseases (CIBERES), Madrid, Spain; 7 Department of Genetics and Microbiology, Universitat Autònoma de Barcelona, Badalona, Spain; 8 University of Vic–Central University of Catalonia (UVic-UCC), Vic, Spain

**Keywords:** Policy and public health in microbiology, Infectious-disease diagnostics

## Abstract

Monitoring the emergence of new SARS-CoV-2 variants is important to detect potential risks of increased transmission or disease severity. We investigated the identification of SARS-CoV-2 variants from real-time reverse transcriptase polymerase chain reaction (RT-PCR) routine diagnostics data. Cycle threshold (Ct) values of positive samples were collected from April 2021 to January 2022 in the Northern Metropolitan Area of Barcelona (*n* = 15,254). Viral lineage identification from whole genome sequencing (WGS) was available for 4618 (30.3%) of these samples. Pairwise differences in the Ct values between gene targets (ΔCt) were analyzed for variants of concern or interest circulating in our area. A specific delay in the Ct of the N-gene compared to the RdRp-gene (ΔCt_NR_) was observed for Alpha, Delta, Eta and Omicron. Temporal differences in ΔCt_NR_ correlated with the dynamics of viral replacement of Alpha by Delta and of Delta by Omicron according to WGS results. Using ΔCt_NR_, prediction of new variants of concern at early stages of circulation was achieved with high sensitivity and specificity (91.1% and 97.8% for Delta; 98.5% and 90.8% for Omicron). Thus, tracking population-wide trends in ΔCt values obtained from routine diagnostics testing in combination with WGS could be useful for real-time management and response to local epidemics.

## Introduction

The severe acute respiratory syndrome coronavirus 2 (SARS-CoV-2) pandemic has been closely monitored by health authorities due to its threat to global public health. For adequate public health planning, it is essential to track the epidemic trajectory and infection incidence in a timely manner^1^. Reverse transcriptase real-time polymerase chain reaction (RT-PCR) testing is widely used for SARS-CoV-2 diagnosis. Testing provides an estimate of the total number of positive cases in the community, which is taken into account in decision-making on health interventions. At the same time, the emergence of novel variants is also closely monitored using whole genome sequencing (WGS). The detection of those recently introduced variants that pose an increased transmission or fatality risk might also affect decision-making^2^. Although WGS is the gold standard for variant identification, it is costly and time-consuming. Therefore, insufficient resources might limit its implementation in low- and middle-income countries, preventing their ability to obtain exhaustive epidemiological knowledge on circulating SARS-CoV-2 variants. RT-PCR provides cycle threshold (Ct) information related to the amount of virus present in the clinical sample that is commonly discarded due to its semiquantitative nature^3^. In the case of SARS-CoV-2, Ct values at the individual-level have been proposed to help in making a decision about patient isolation^4,5^ and assessing disease severity^6,7^. However, the high variability that can arise from testing across different platforms, sample collection procedures, as well as the differences between asymptomatic and presymptomatic infections and the impact of host characteristics on SARS-CoV-2 viral kinetics has limited the usefulness of individual Ct evaluation. At the population level, Ct data has been suggested to be useful to predict epidemic trajectory^3^ and to detect those individuals with higher probability of being superspreaders of the virus^8^. In certain cases, Ct data can also provide information on the variant present in the sample as the accumulated genetic changes may cause diagnostic failure. For example, variants B.1.1.7 (Alpha) and BA.1 (Omicron) were characterized by S-gene target failure (SGTF) for at least one RT-PCR assay due to deletion of amino acids 69 and 70 (Δ69-70) in the spike gene, which has been usefully used to monitor the spread of these two variants^9–11^. However, viral mutations do not necessarily imply complete target failure. Amplification primers and/or probe binding affinity to target DNA can be modified by arising mutations in several manners that can decrease target amplification efficiency, which can be indirectly observed by higher Ct values of the affected target. In this vein, it has been proposed that the Allplex™ SARS-CoV-2/FluA/FluB/RSV Assay can use N-gene target data as a proxy to detect the Alpha variant of concern, which is characterized by an increase > 8 in the Ct value for the N-gene target (Ct_N_) compared to the Ct value for the S-gene target (Ct_S_)^8^. Other RT-PCR strategies to predict variant presence have also been developed, such as assays that detect lineage-specific mutations. However, the implementation of these so-called variant kits usually implies an additional RT-PCR test after initial diagnostics, increasing the sample-processing burden of clinical laboratories.

Here we analyzed Ct differences among different SARS-CoV-2 genetic targets in samples collected for routine RT-PCR diagnostics from April 2021 to January 2022 in the Northern Metropolitan Area of Barcelona. Using WGS results as gold standard, we analyzed the utility of Ct differences to detect viral transitions and predict the viral variant present in the sample during such transition periods. Insights into the dynamics of the viral replacements of Alpha by Delta and of Delta by Omicron in that geographical area are provided. Importantly, a new metric to monitor the pandemic in almost real time based on the analysis of the temporal evolution of differences among Ct targets is presented.

## Results

### Characteristics of the study samples

**Figure 1:**
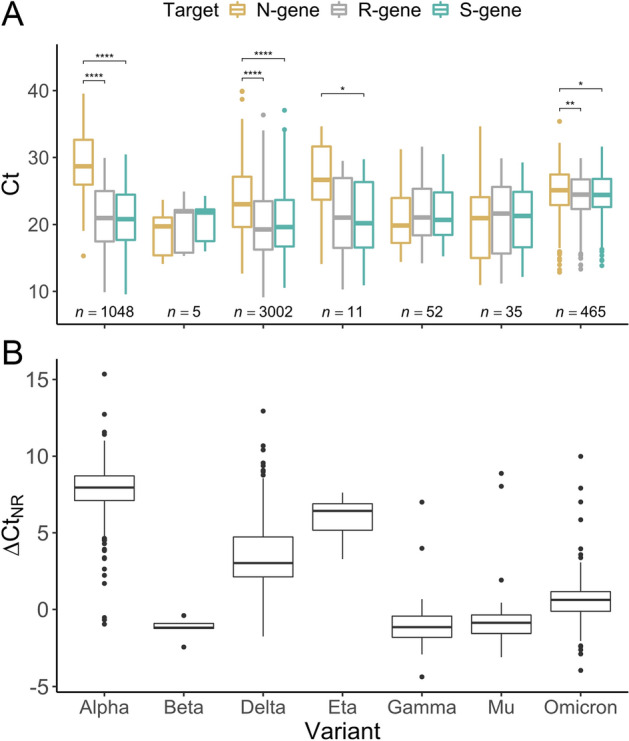
Characteristic RT-PCR profiles identify multiple SARS-CoV-2 variants. (**A**) Ct distributions for each gene target (N, R and S) for variants Alpha, Beta, Delta, Eta, Gamma, Mu and Omicron. Wilcoxon signed-rank test. *: *p*-value < 0.05. **: *p*-value < 0.01. ****: *p*-value < 0.0001. (**B**) Distributions of differences between Ct_N_ and Ct_R_ (∆Ct_NR_) for each variant.

Routine high-quality Ct data was obtained for 15,254 samples from patients tested for SARS-CoV-2 at a reference laboratory. Median age was 36 years old (interquartile range 19–53) and 53.5% samples belonged to female individuals. Among them, 4618 samples (30.3%) had been additionally processed by WGS according to several indications for sequencing in the context of the regional genomic surveillance program^16^ and had a lineage assigned. Identified lineages corresponded to five current or former variants of concern and two variants of interest, as of March 2022 (for a distribution of the number of cases for each variant see Fig. 1A).

### Identification of variants using Ct data

Pairwise differences in the Ct values between gene targets (ΔCt) were analyzed for variants that circulated in our area from April 2021 to January 2022. Variants Beta, Gamma and Mu had similar Ct_S_, Ct_N_ and Ct_R_ values with no significant pairwise differences. Variant Eta had a significantly higher Ct_N_ compared to Ct_S_ (26.9 vs 20.7, *p*-value < 0.05) but the difference was not statistically significant between Ct_N_ and Ct_R_ (26.9 vs 21.1, *p*-value = 0.065). Alpha, Delta and Omicron presented a delayed detection of N-gene target indicated by a significantly higher Ct_N_ compared to both Ct_R_ and Ct_S_ (N-gene *vs*. R-gene/S-gene: 29.1 *vs*. 21.2/21.0, *p*-value < 0.0001; 23.4 *vs*. 20.0/20.3, *p*-value < 0.0001; 24.8 *vs*. 24.2/24.4, *p*-value < 0.01/*p*-value < 0.05; respectively), (Fig. 1). Finally, no significant differences were observed between Ct_R_ and Ct_S_ for any of the assessed variants. Based on these observations, we hypothesized that ∆Ct_NR_ could be a valuable metric to detect transitions between viral variants. From this perspective, multivariate linear regression was performed, showing that viral variant had a significantly effect on ∆Ct_NR_ while age and gender did not (See Supplementary Figs. 1 and 2 for ∆Ct_NR_ distributions for age and sex). Significant differences (Tukey's ‘Honest Significant Difference’ method) were observed for all pairwise variant comparisons except for Beta vs. Gamma/Mu/Omicron and Mu vs. Gamma (Supplementary Table 1). Additionally, we tested whether any trends existed between overall viral load, proxied by individual Ct values (Ct_N_, Ct_R_ and Ct_S_), and ∆Ct_NR_ and did not find any strong correlations (Supplementary Fig. 3).

### Detection of viral variant transitions using temporal ∆Ct_NR_ trends

**Figure 2:**
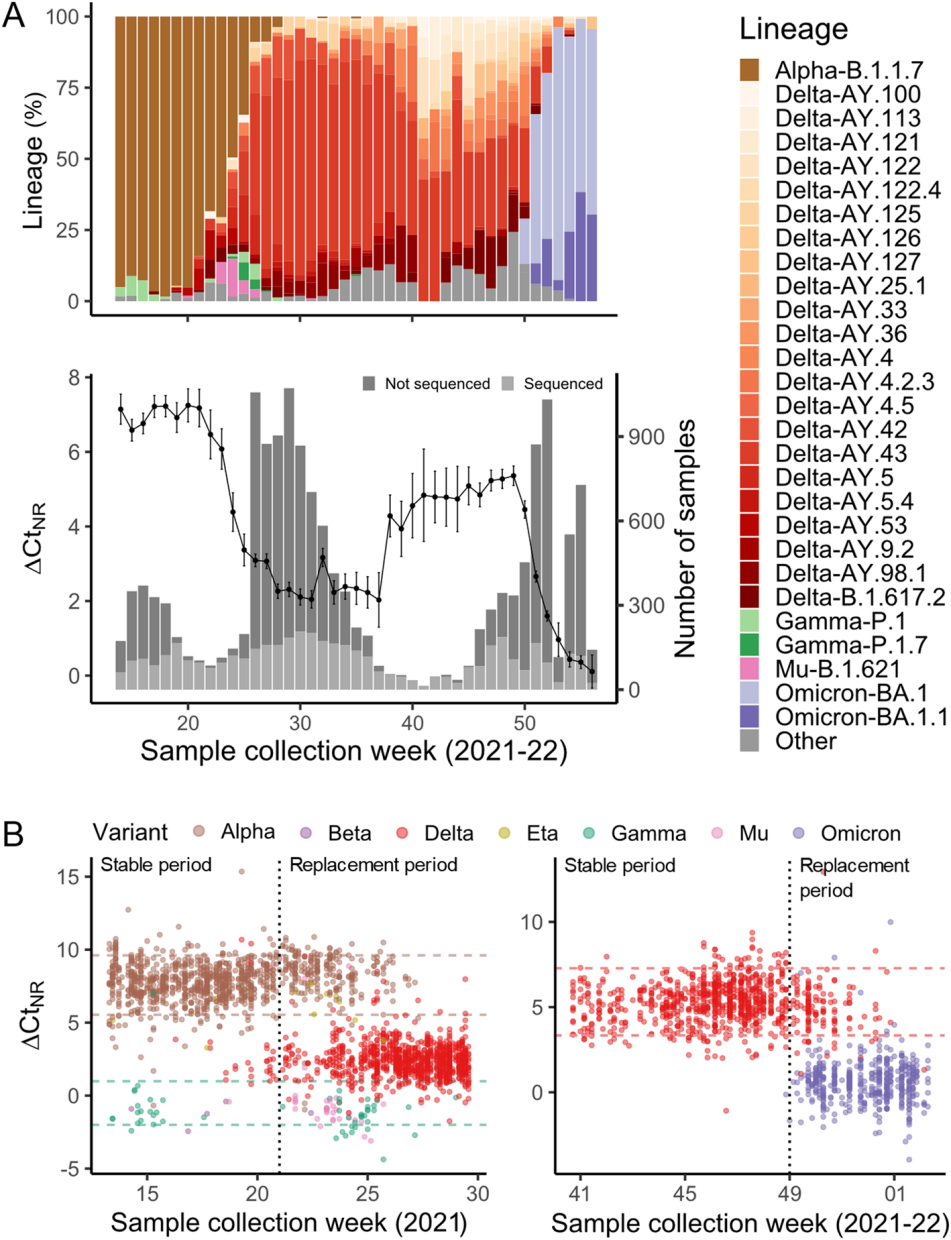
Temporal evolution of ∆Ct_NR_ registered viral variant replacements. (**A**) Top: Weekly temporal evolution of SARS-CoV-2 lineages as per WGS results. Bottom: Weekly number of high-quality RT-PCR samples are indicated with bars (shades of grey indicate availability of WGS results). Weekly temporal evolution of ∆Ct_NR_ is depicted by a line-dot trend with standard error shown in bars. (**B**) Models for variant classification during Alpha-Delta transition (left) and Delta-Omicron transition (right). Data used for model construction is shown in the stable period while data used for model evaluation is used on the replacement period. Each sub-dataset is separated by a vertical dotted line. Cut-off values determined at the 5th and 95th percentile values of circulating variants during the stable period are indicated by horizontal dashed lines colored according to each variant.

To estimate the possibility to detect replacements between viral variants from routine RT-PCR diagnostics data, we evaluated the population-wide weekly evolution of ∆Ct_NR_ and compared it to WGS data (Fig. 2A). WGS results detected the viral replacement of Alpha by Delta occurring between weeks 19–28, and replacement of Delta by Omicron started at week 50 and continued until week 3 of 2022 (Fig. 2A, bottom). Weekly temporal evolution of ∆Ct_NR_ over the study period evidenced three significant changes: a decreasing trend in ∆Ct_NR_ was observed between weeks 20 and 30, ∆Ct_NR_ then increased during weeks 37–41 and finally decreased again after week 49. The first and third periods of ∆Ct_NR_ changes closely matched the Alpha-Delta and Delta-Omicron viral transitions, respectively, while the second period preceded a change in the prevalence of specific Delta sublineages when a sharp increase in AY.122 prevalence was observed during a period of low incidence.

In order to identify which mutations could be related with the observed changes in ∆Ct_NR,_ we scanned for mutations in the vicinity of the N-gene start codon (nucleotide position 28,274). In the scanned region (nucleotide positions 28,150–28,500), we observed eleven unique combinations of certain deletions (del2874, del2878-81, del28362-69/70), and/or substitutions (A28271T, GAT28280CTA, A28299T, C28308G, C28311T, and A28461G) that appeared in a variant-specific manner in the analyzed sequences. Thus, these nine genetic changes could potentially be involved in the observed Ct_N_ delays. Variants Beta, Gamma and Mu presented none of these mutations and had a low ∆Ct_NR_ (− 0.8 ± 1.9); therefore, this group of three variants was used as the reference profile to determine that in nine out of the eleven unique combinations of mutations detected, the mutation profile was associated with a significant increase in ∆Ct_NR_ ranging from 0.59 ± 1.24 for the profile combining A28271T, C28311T, and del28362-69/70, which was specific for Omicron, to 7.9 ± 1.3 for the profile combining del2874 and GAT28280CTA, which was specific for Alpha (Supplementary Fig. 4).

### Identification of viral variant during replacement periods

The observation of stable and variable periods during the temporal evolution of ∆Ct_NR_ (Fig. 2A, bottom) lead to the hypothesis that newly introduced variants presenting a different ∆Ct_NR_ could be readily identified during variant replacement periods. Given the uncertainty of the ∆Ct_NR_ value of future variants, we classified as a potentially new variant any sample that displayed a ∆Ct_NR_ value outside of the 5th–95th percentile range observed for the circulating variant/s during the previous stable period. Thus, here we used WGS data to build two prediction models and evaluate their performance to detect samples containing the newly introduced variant. Particularly, we assessed the ability of the models to detect Delta during the Alpha-Delta transition (model 1) and Omicron during the Delta-Omicron transition (model 2), respectively.

For model 1, samples from the beginning of the study period (week 14) until week 20 (stable period, *n* = 751) were used to determine the 5th and 95th percentile of ∆Ct_NR_ for Alpha (5.54, 9.61), which was predominant during this period, and Gamma (− 2.00, 0.99), which circulated at a much lower frequency. These cut-off values were then used during weeks 21–30 (replacement period, *n* = 1349) to predict the presence of the newly introduced Delta variant in samples with intermediate ∆Ct_NR_ values between Gamma and Alpha (0.99 < ∆Ct_NR_ < 5.54), as observed by the first available WGS results for Delta. This model showed 91.1% sensitivity and 97.8% specificity for the identification of Delta (Fig. 2B, left). However, the model was not able to distinguish Mu from Gamma as these variants presented similar ∆Ct_NR_ populations. The overall accuracy was 86.7% (95%CI, 84.8–88.5) and the Kappa statistic was 0.729 (Supplementary Table 2), being these statistics slightly lower in the Ct_R_ range > 25 (Supplementary Fig. 5).

Similarly, for model 2, the 5th and 95th percentile of ∆Ct_NR_ for the circulating Delta lineages during weeks 41–48 (stable period, *n* = 731) were established at 3.34 and 7.28, respectively. From week 49 of 2021 to week 3 of 2022 (replacement period, *n* = 628), model 2 predicted Omicron in samples with ∆Ct_NR_ < 3.34 with 98.5% sensitivity and 90.8% specificity (Fig. 2B, right). The resulting overall accuracy for model 2 was 95.5% (95%CI, 93.6–97.0) while the Kappa statistic was 0.883 (Supplementary Table 3). In this case, optimal accuracy and Kappa values were observed in the Ct_R_ range between 15 and 25 (Supplementary Fig. 5).

## Discussion

Routine diagnostics using RT-PCR are usually interpreted as either positive or negative while Ct information is commonly disregarded. Here, we show how differences in the Ct values of gene targets could be used to detect SARS-CoV-2 viral variant replacements and, in certain cases, to infer the presence of a new variant in the sample. Regarding the observed delay in the N gene, we provide new data for Eta, Mu and Omicron variants and extend the existing evidence for the shift in gene N compared to both gene R and gene S for variants Alpha, Beta, Delta and Gamma. Giovacchinni et al*.*^12^ reported ∆Ct_NS_ values similar to the ∆Ct_NR_ values reported here for variants Alfa, Delta and Gamma. Our results also qualitatively agree with those published by Wollschläger et al.^17^ for the Alpha variant.

Previous studies have demonstrated the usefulness of collecting and analyzing Ct data obtained by routine RT-PCR. At the population level, Ct data has been used to infer population-wide viral load kinetics and epidemic trajectory^3,8^ and to track the prevalence of Alpha through the change in the proportion of samples showing SGTF^9^. Contrarily, the high variability of Ct values due to various reasons has limited the usefulness of clinical evaluation of individual Ct values. However, here we used differences between Ct values of different targets (∆Ct) for each sample, which we showed are variant-specific and narrower than absolute Ct distributions (Fig. 1). Therefore, we hypothesize that the effect of individual sample characteristics influencing absolute Ct values are dissipated when using ∆Ct values instead and showed that ΔCt_NR_ has a fairly constant value over a wide range of absolute Ct values (Supplementary Fig. 3). Nevertheless, overall, optimal method performance was observed for Ct_R_ values ≤ 25 (Supplementary Fig. 5). Here we demonstrate that transition between specific viral variants could be evaluated in a rapid manner by analyzing the temporal evolution of ∆Ct_NR_. Similarly, a recent study by Valley-Omar et al.^18^ showed the ability to detect the replacement of variant Beta by Delta occurred during May–July 2021 in South Africa by tracking the Ct differences between gene R and gene E using the Allplex 2019-nCoV assay. Our data in combination with that provided by Valley-Omar et al., is an indication that the methodology presented here could be valuable for various commercially available SARS-CoV-2 diagnostics kits. Nevertheless, certain variant transitions could be missed by this methodology if there is a lack of ∆Ct_NR_ change between the replaced and replacing variants, for example as in the hypothetical case of a replacement between variants Gamma, Beta and Mu. Therefore, WGS will remain necessary to assess the utility and performance of the methodology presented here for the detection of future variants.

Genomic surveillance programs for SARS-CoV-2 were rapidly implemented in multiple countries shortly after the COVID-19 pandemic started. Despite great effort, sequencing capabilities are limited and vary within country regions and between countries^19^, sometimes restricting the maximum number of positive RT-PCR samples that can be used for genomic surveillance. Therefore, sequencing results might be available only for a reduced percentage of the total number of cases, especially during periods of elevated incidence, as exemplified in the current study (Fig. 2A, bottom). Contrarily, during periods of low incidence, the reduced number of samples might cause a delay in sequencing results owing to the need to accumulate samples for proper cost-effectiveness optimization of each sequencing run. Regardless of incidence, Ct data offers the possibility to monitor a large fraction of positive samples, without additional costs over diagnostic RT-PCR and in a faster manner than WGS. At the same time, Ct monitoring can reduce underlying systematic biases in sample selection for genomic surveillance. It is also important to remark that sustainability of high levels of sequencing might be compromised and some countries are already undergoing a change in their genomic surveillance programs for SARS-CoV-2, decreasing their sequencing efforts and focusing them on highly vulnerable populations or serious COVID-19 cases based on the premise that a higher proportion of immunized individuals has been reached^20,21^. Thus, the new strategy presented here to track changing trends in ΔCt among RT-PCR targets in combination with available genomic surveillance using WGS could be helpful for real-time epidemics management and public health response.

This study has limitations. Firstly, as low viral loads (higher Ct values) might lead to failed amplification and prevent the calculation of the differences observed among Ct targets, future analysis should only be applied to samples with detection of all gene targets, as it was done in this study. Moreover, model performance could not be inferred for Ct_R_ > 30 because samples with this characteristic were scarce in our dataset as WGS often yields bad quality sequences in this Ct range. Therefore, the methodology presented here should ideally applied to samples with high rather than low viral loads. Secondly, our study is limited by the proprietary character of the Allplex™ SARS-CoV-2/FluA/FluB/RSV Assay. Since the genomic region targeted by the assay primers and probes is protected, we were unable to confirm that the mutations correlating with the delay in ∆Ct_NR_ are actually responsible for these changes. Finally, it is important to remark that the novel tool presented here will need to be properly reevaluated and updated according to newly arising viral variants and their characterization by WGS.

In conclusion, our results demonstrate that Ct differences between gene targets from routine molecular diagnostics can be used to monitor replacements between SARS-CoV-2 variants. This new simple metric would allow local epidemic monitoring in almost real time and inform response decisions.

## Methods

### Study design

High-quality RT-PCR Ct data, defined by detection of all gene targets, was retrospectively obtained from the laboratory information system of a reference hospital for a total of 15,254 positive samples collected for routine diagnostics between April 6th, 2021, and January 18th, 2022. Among them, an assigned viral lineage provided by WGS was available for 4618 samples (30.3%).

### Sample collection and molecular diagnosis of SARS-CoV-2

Naso-pharyngeal and nasal swabs were collected by trained personnel and used to detect the presence of SARS-CoV-2 infection using RT-PCR. RNA was extracted using the STARMag reagent (Seegene) on a Microlab Starlet IV (Hamilton Life Science Robotics) automatic extractor. The presence of SARS-CoV-2 was confirmed by RT-PCR in a single step with the Allplex™ SARS-CoV-2/FluA/FluB/RSV Assay (Seegene, Cat No. RV10259X) on CFX96 instruments (BIO-RAD).

### SARS-CoV-2 whole genome sequencing (WGS)

For WGS, SARS-CoV-2 positive samples with Ct ≤ 30 for R-gene target were selected to maximize sequencing success rate. Extracted RNA stored at – 80 °C was reverse-transcribed to cDNA with SuperScript IV (Invitrogen) and random hexamers, according to the manufacturer's protocol. Then, the whole genome of SARS-CoV-2 was amplified using the ARTIC network v3 amplicon panel (Integrated DNA Technologies). Purified DNA was processed with either the Illumina DNA Prep kit or the Nextera XT kit for the preparation of dual-indexed libraries (Illumina) and sequenced on an Illumina MiSeq platform.

### Bioinformatics analysis of WGS data

Raw data analysis was performed using viralrecon pipeline^13^. Sequence reads were quality-filtered and adapter primer sequences were trimmed using Trimmomatic^14^. Sequencing reads were then aligned against the reference Wuhan/Hu-1/20219 variant (NCBI accession number: NC_045512.2) using the bowtie2 tool^15^, while consensus genomic sequence was called from the resulting alignments using iVar software at the 25% threshold. SARS-CoV-2 lineages were assigned using Pangolin version v.3.1.20 (using PANGO version v.1.2.127 and pangoLEARN version of 28-02-2022).

### Statistical analyses and variant classification model

The stats package within R (version 4.1.0) was used to: (1) detect statistical differences among all possible pairwise combinations of Ct values for genes N, S and R were evaluated using the Wilcoxon signed-rank test, (2) detect statistical differences for ΔCt_NR_ distributions among all possible pairwise combinations of variants using the TukeyHSD function, and (3) perform multivariate linear regression using the lm function. For each period of viral variant replacement observed, a classification model was built. Using WGS as the gold standard for variant identification, cut-offs for values for ∆Ct_NR_ were established at the 5th and 95th percentiles for circulating variants observed during the previous weeks. Each model classifies samples outside the 5th–95th percentile range as potentially new variants.

### Ethical approval

WGS data collection was done as part of SARS-CoV-2 public health surveillance. As result from this surveillance activity, SARS-CoV-2 sequences had been made publicly available through the GISIAID initiative (www.gisaid.org). Retrospective Ct data from diagnostic routine SARS-CoV-2 RT-PCR testing was anonymized before analysis.

## Supplementary Information

Supplementary Information 1.

Supplementary Table 4.

## Data Availability

Data is available as Supplementary Table 4.

## Abbreviations

Ct: Cycle threshold
Ct_N_: Cycle threshold for gene N
Ct_R_: Cycle threshold for gene RdRP
Ct_S_: Cycle threshold for gene S
RT-PCR: Real-time reverse transcriptase polymerase chain reaction
SGTF: S-gene target failure
WGS: Whole genome sequencing
ΔCt: Difference in Ct values between gene targets
ΔCt_NR_: Ct_N_−Ct_R_
ΔCt_NS_: Ct_N_−Ct_S_
ΔCt_RS_: Ct_R_−Ct_S_
