# Supplementary material for: Monitoring SARS-CoV-2 variant transitions using differences in diagnostic cycle threshold values of target genes
Source: Sci Rep. 2022 Dec 17;12:21818. doi: 10.1038/s41598-022-25719-9 (PMC9758454; doi:10.1038/s41598-022-25719-9)
Supplement: Supplementary file 1 — Supplementary Information 1. [file 41598_2022_25719_MOESM1_ESM.docx]

**Supplementary Table 1.** Pairwise variant comparisons for ΔCt_NR_.

| Comparison | Difference | 95% CI | Adj. *p*-value |
| --- | --- | --- | --- |
| Beta-Alpha | -9.14 | (-11.30, -6.99) | <0.001 |
| Delta-Alpha | -4.38 | (-4.55, -4.21) | <0.001 |
| Eta-Alpha | -1.99 | (-3.45, -0.54) | <0.01 |
| Gamma-Alpha | -8.87 | (-9.55, -8.19) | <0.001 |
| Mu-Alpha | -8.27 | (-9.10, -7.45) | <0.001 |
| Omicron-Alpha | -7.14 | (-7.40, -6.87) | <0.001 |
| Delta-Beta | 4.76 | (2.61, 6.91) | <0.001 |
| Eta-Beta | 7.15 | (4.56, 9.74) | <0.001 |
| Gamma-Beta | 0.27 | (-1.97, 2.52) | 1 |
| Mu-Beta | 0.87 | (-1.43, 3.16) | 0.923 |
| Omicron-Beta | 2.01 | (-0.15, 4.17) | 0.088 |
| Eta-Delta | 2.39 | (0.94, 3.84) | <0.001 |
| Gamma-Delta | -4.49 | (-5.16, -3.82) | <0.001 |
| Mu-Delta | -3.89 | (-4.71, -3.08) | <0.001 |
| Omicron-Delta | -2.76 | (-2.99, -2.52) | <0.001 |
| Gamma-Eta | -6.88 | (-8.47, -5.29) | <0.001 |
| Mu-Eta | -6.28 | (-7.94, -4.62) | <0.001 |
| Omicron-Eta | -5.15 | (-6.61, -3.68) | <0.001 |
| Mu-Gamma | 0.60 | (-0.45, 1.64) | 0.634 |
| Omicron-Gamma | 1.73 | (1.03, 2.44) | <0.001 |
| Omicron-Mu | 1.14 | (0.30, 1.98) | <0.01 |

**Supplementary Table 2.** Confusion matrix with summary of classification statistics to estimate performance of model 1.

**Model 1.** Accuracy (95%CI): 86.7% (84.8-88.5). Kappa statistic: 0.729

|  |  | **WGS results** | | | | | | | | |
| --- | --- | --- | --- | --- | --- | --- | --- | --- | --- | --- |
|  |  | Alpha | Beta | Delta | Eta | Gamma | Mu | PNV1 | PNV2 | Total |
| **Model classification** | Alpha | 288 | 0 | 15 | 6 | 0 | 2 | 0 | 0 | 311 |
|  | Beta | 0 | 0 | 0 | 0 | 0 | 0 | 0 | 0 | 0 |
|  | Delta | 6 | 0 | 860 | 2 | 0 | 1 | 0 | 0 | 869 |
|  | Eta | 0 | 0 | 0 | 0 | 0 | 0 | 0 | 0 | 0 |
|  | Gamma | 2 | 1 | 68 | 0 | 22 | 27 | 0 | 0 | 120 |
|  | Mu | 0 | 0 | 0 | 0 | 0 | 0 | 0 | 0 | 0 |
|  | PNV1 | 36 | 0 | 1 | 0 | 0 | 0 | 0 | 0 | 37 |
|  | PNV2 | 0 | 0 | 0 | 0 | 9 | 3 | 0 | 0 | 12 |
|  | Total | 332 | 1 | 944 | 8 | 31 | 33 | 0 | 0 | 1349 |

PNV1: Potentially new variant 1 (ΔCt_NR_>Alpha’s ΔCt_NR_ 95^th^ percentile). PNV2: Potentially new variant 2 (ΔCt_NR_<Gammas’s ΔCt_NR_ 5^th^ percentile).

**Supplementary Table 3.** Confusion matrix with summary of classification statistics to estimate performance of model 2.

**Model 2.** Accuracy (95%CI): 95.5% (93.6-97.0). Kappa statistic: 0.883

|  |  | **WGS results** | | | |
| --- | --- | --- | --- | --- | --- |
|  |  | Delta | Omicron | PNV3 | Total |
| **Model classification** | Delta | 142 | 5 | 0 | 147 |
|  | Omicron | 15 | 458 | 0 | 473 |
|  | PNV3 | 6 | 2 | 0 | 8 |
|  | Total | 163 | 465 | 0 | 628 |

PNV3: Potentially new variant 3 (ΔCt_NR_>Delta’s ΔCt_NR_ 95^th^ percentile).


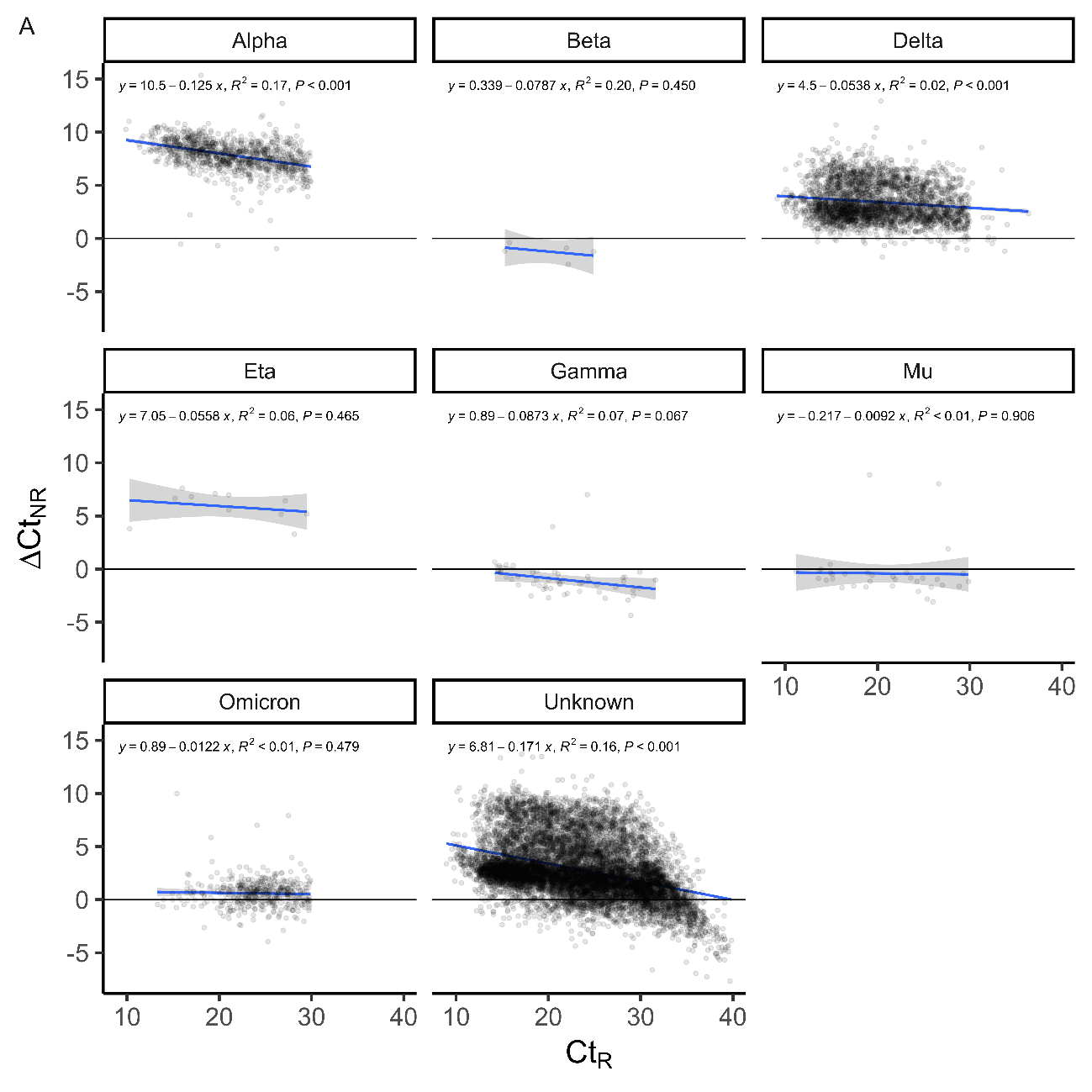


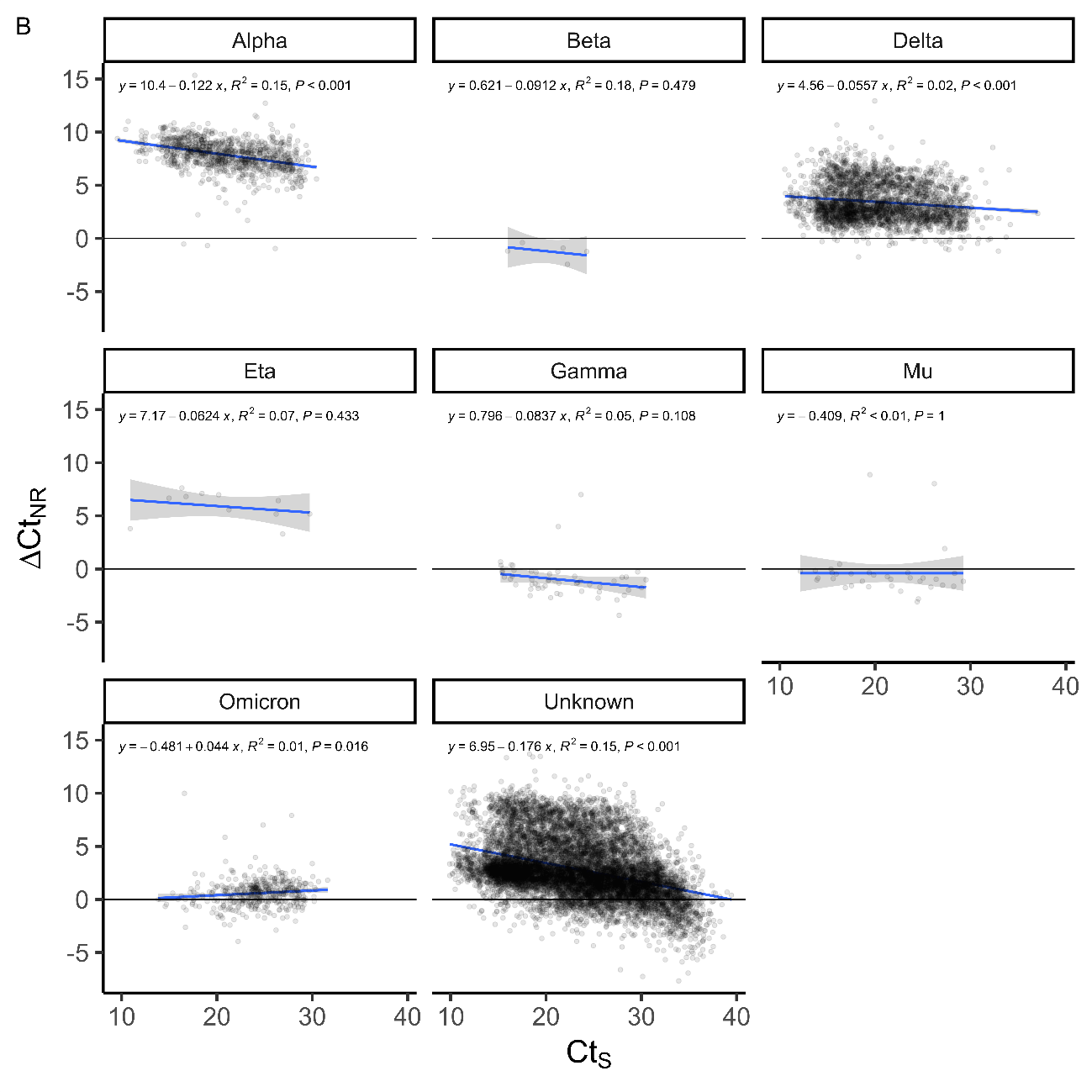


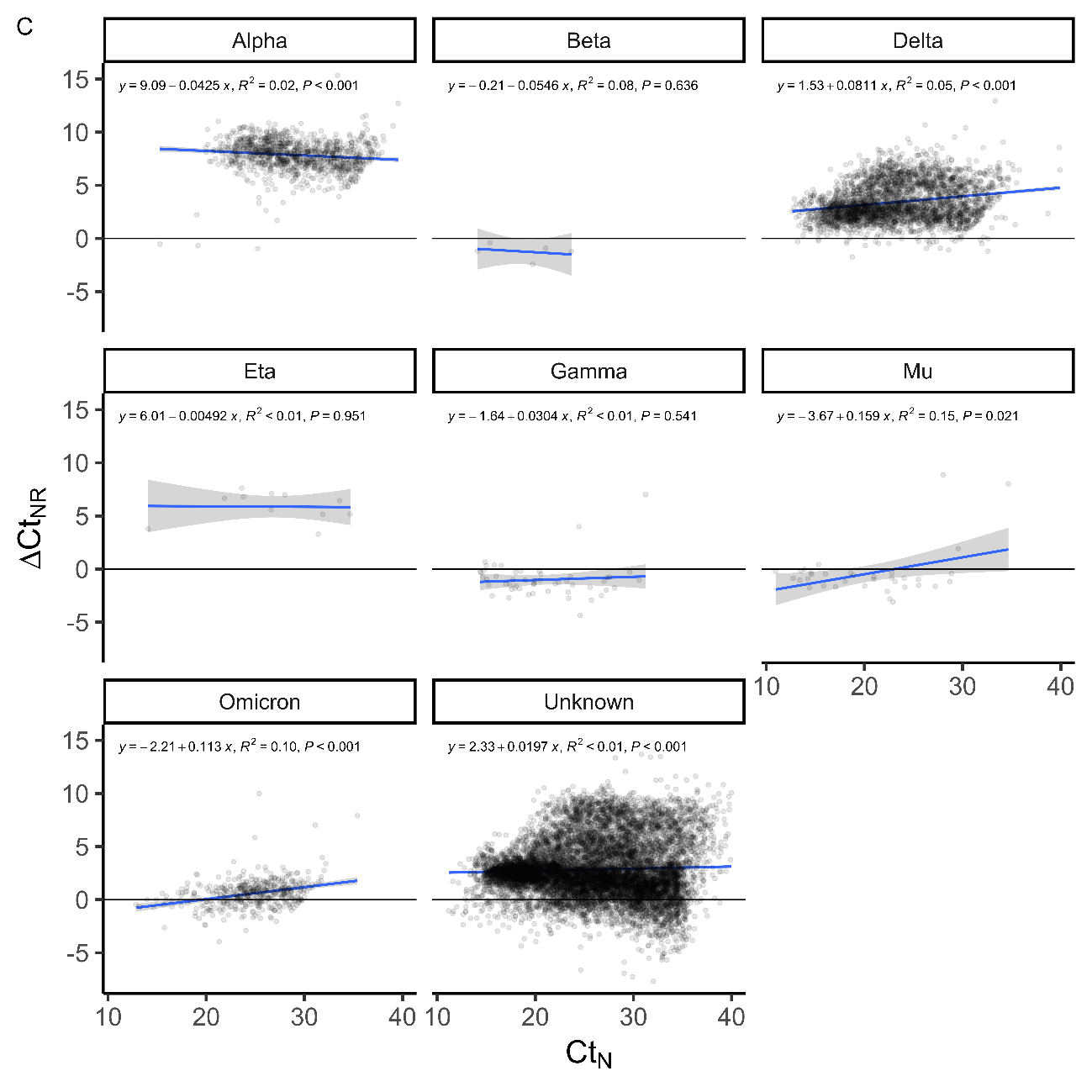


**Supplementary Figure 1.** Distribution of ΔCt_NR_ for absolute values of (**A**) Ct_R_, (**B**) Ct_S_, and (**C**) Ct_N_. Linear regression equation, Pearson’s R^2^ values and *p*-values are presented for each variant as well as all samples that did not undergo whole-genome sequencing (Unknown). Please note that as Ct_R_/Ct_S_ approach the value of 40, the variants with greater ΔCt_NR_, such as Alpha and Delta, are excluded from the dataset as lack of detection of gene N by the PCR assay prevents proper ΔCt_NR_ calculation, potentially biasing regression lines towards negative slopes.

**
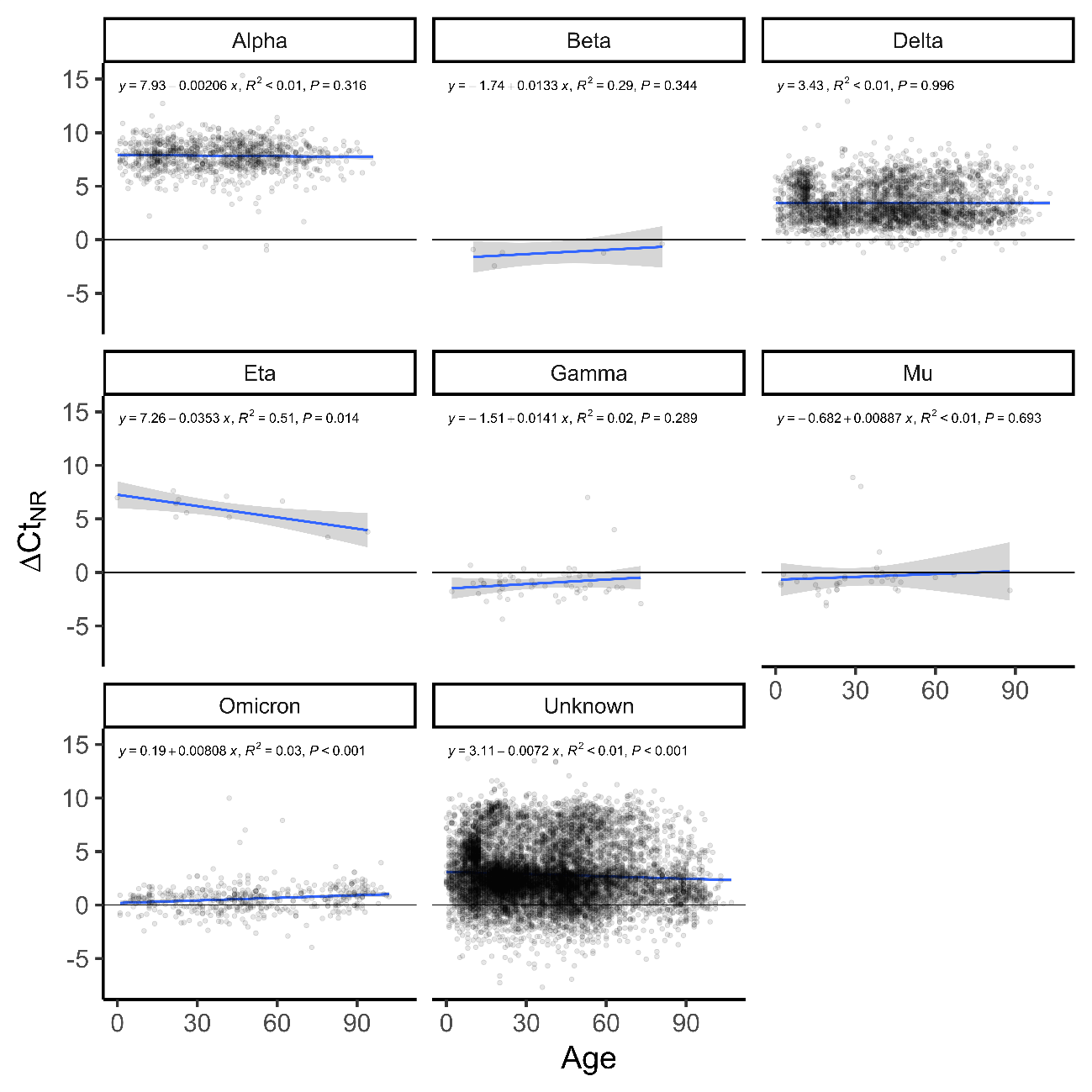
Supplementary Figure 2.** Distribution of ΔCt_NR_ for age. Linear regression equation, Pearson’s R^2^ values and *p*-values are presented for each variant as well as all samples that did not undergo whole-genome sequencing (Unknown).

**
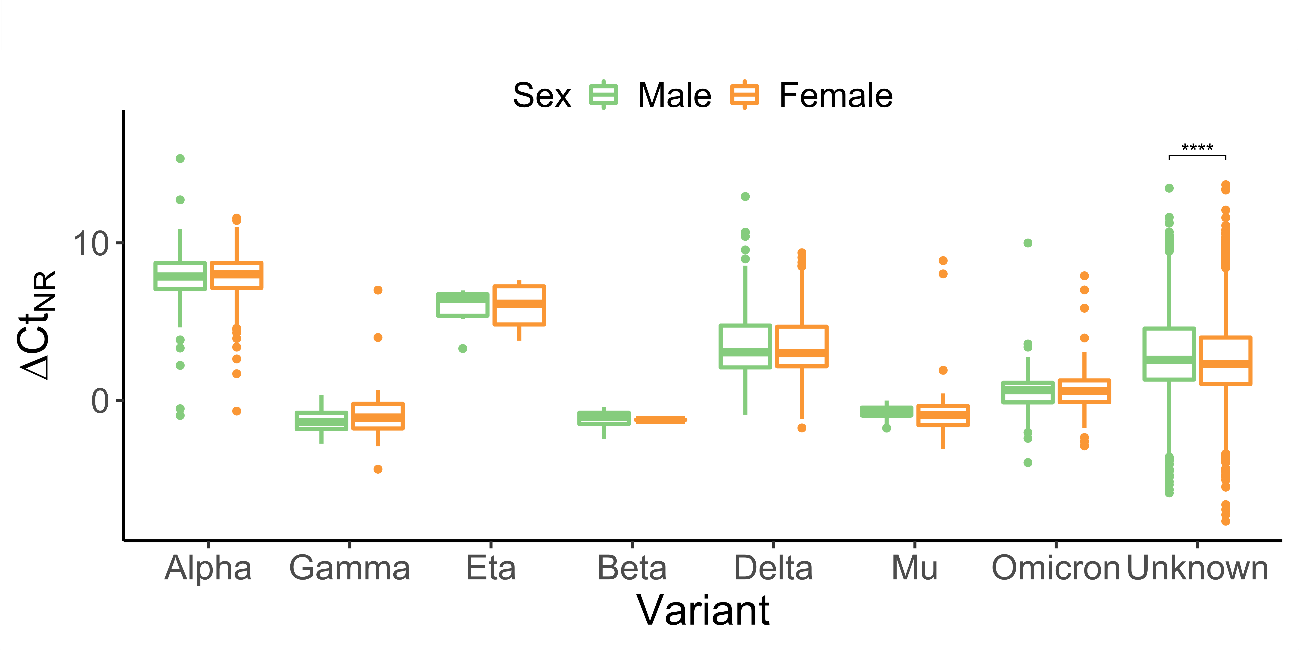
Supplementary Figure 3.** Distribution of ΔCt_NR_ for sex. Linear regression equation, Pearson’s R^2^ values and *p*-values are presented for each variant as well as all samples that did not undergo whole-genome sequencing (Unknown). Significant differences between males and females (Wilcoxon signed-rank test) are indicated (****: p‑value<0.0001).

**
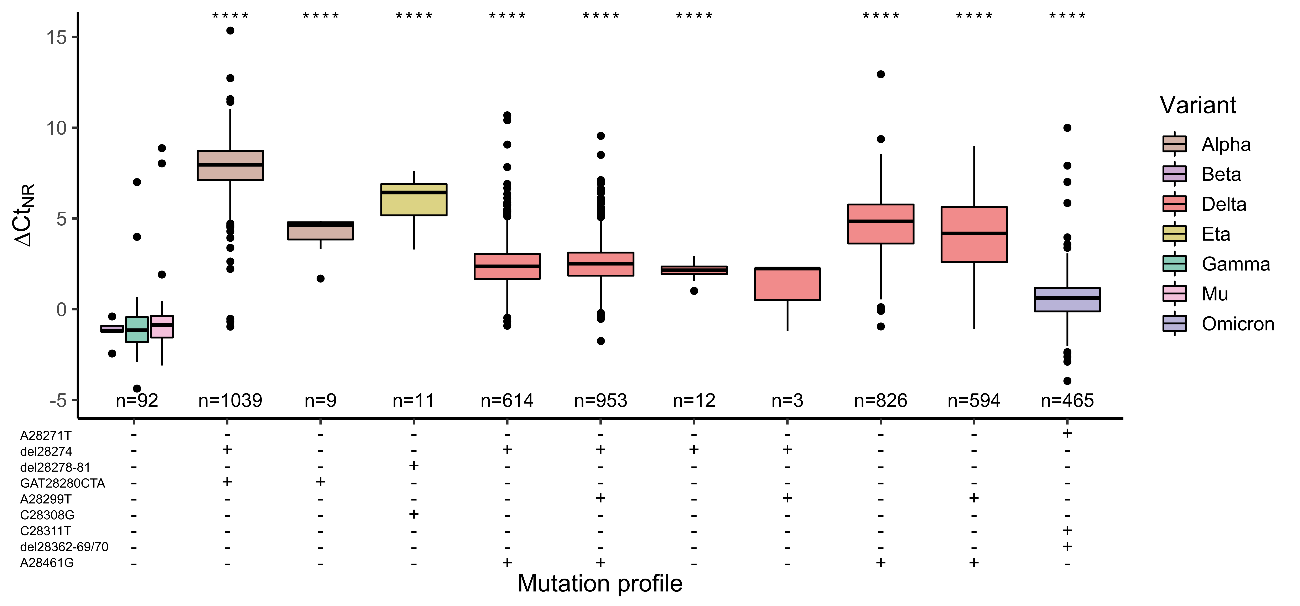
Supplementary Figure 4.** Distribution of ΔCt_NR_ for various mutation profiles. All mutations profiles were present in only one variant except for the reference profile (no mutations detected), which was observed for Beta, Gamma and Mu variants. Significant differences against the Beta/Gamma/Mu reference group (Wilcoxon signed-rank test) are indicated (****: p‑value<0.0001).


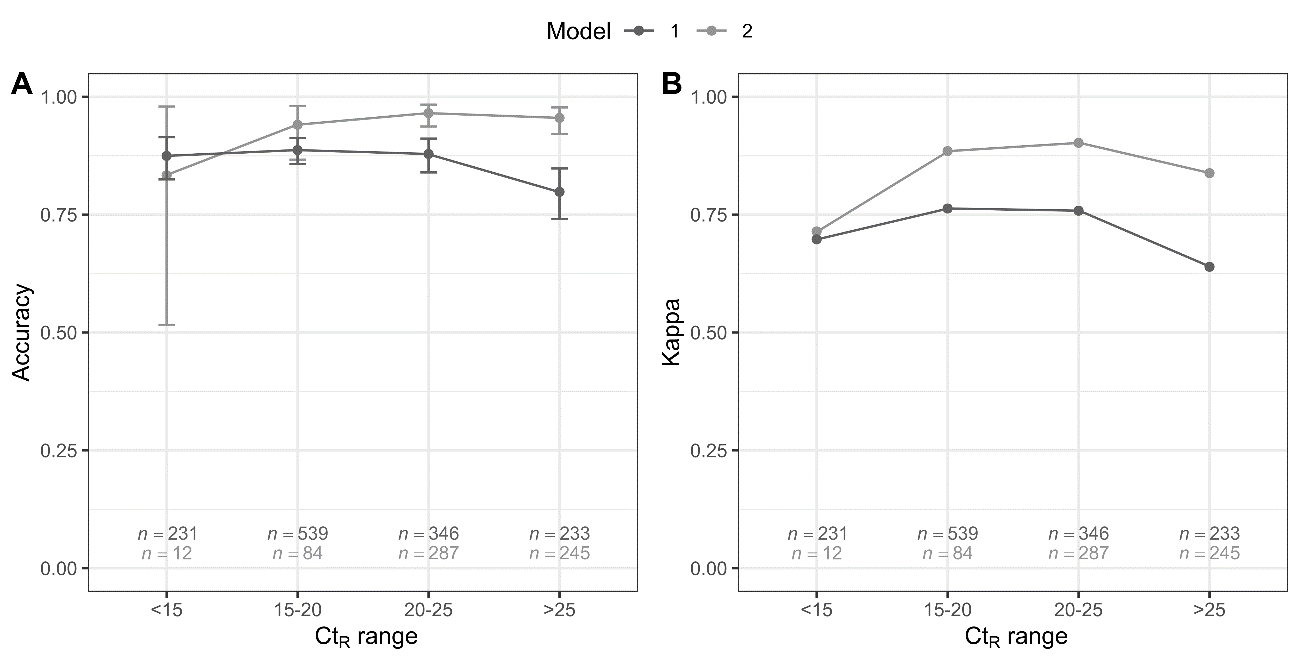
 **Supplementary Figure 5.** Influence of viral load (proxied by the absolute value of Ct_R_) on the accuracy and Kappa statistic values for models 1 and 2. In panel **A**, errors bars represent 95% confidence intervals for accuracy.
